# Usefulness of presepsin in the diagnosis of sepsis in acute kidney injury patients

**DOI:** 10.1186/cc11974

**Published:** 2013-03-19

**Authors:** Y Nakamura, H Ishikura, T Nishida, Y Kawanno, R Yuge, R Ichiki, A Murai

**Affiliations:** 1 Fukuoka University, Fukuoka, Japan

## Introduction

The level of presepsin is useful for differentiating sepsis from noninfectious systemic inflammatory response syndrome. It has been reported that the presepsin levels in patients with chronic renal failure are abnormally high. However, there are no studies investigating the usefulness of presepsin for diagnosis of sepsis in patients with acute kidney injury (AKI). Our purpose of this study is to clarify the diagnostic accuracy of presepsin in patients with AKI and the relationship between presepsin level and AKI severity.

## Methods

This study was conducted as a single-center retrospective study. Blood samples were collected from patients admitted to the emergency room at Fukuoka University Hospital between June 2010 and October 2012. We enrolled 254 patients with suspected sepsis and other disease patients. We classified the patients into an AKI group according to the RIFLE criteria (Risk *n *= 52, Injury *n *= 39, Failure *n *= 41, Loss of kidney function and End-stage kidney disease *n *= 7) and a non-AKI group (*n *= 115). The AKI patient group was further classified into a sepsis group and a nonsepsis group in each AKI stage and we analyzed the diagnostic accuracy of presepsin in patients with sepsis.

## Results

For the non-AKI patients, the median of presepsin in patients with nonsepsis (*n *= 78) and the sepsis group (*n *= 37) were 406 pg/ml (range: 86 to 4,374) and 1,065 pg/ml (range 86 to 9,960), respectively (*P *< 0.0001). For the Risk patients, the median of presepsin in patients with nonsepsis (*n *= 25) and the sepsis group (*n *= 27) were 299 pg/ml (range: 71.2 to 3,361) and 831 pg/ml (range: 233 to 16,759), respectively (*P *< 0.01). For the Injury patients, the median of presepsin in patients with nonsepsis (*n *= 12) and the sepsis group (*n *= 27) were 463 pg/ml (range: 122 to 1,197) and 1,451 pg/ml (range: 237 to 4,200), respectively (*P *< 0.001). For the Failure patients, the median of presepsin in patients with nonsepsis (*n *= 14) and the sepsis group (*n *= 27) were 1,607 pg/ml (range: 454 to 8,516) and 1,523 pg/ml (range: 293 to 16,764), respectively (*P *= 0.175). The diagnostic accuracy of presepsin in patients with sepsis was determined by ROC analysis, the area under the curve was 0.789 for the non-AKI patient group, 0.735 for the Risk patient group, 0.855 for the Injury patient group and 0.593 for the Failure patient group.

## Conclusion

In Failure and more progressed AKI patients, the diagnostic accuracy of the presepsin level was lower than the other groups.
